# Supplementary material for: ER and SOCE Ca2+ signals are not required for directed cell migration in human iPSC-derived microglia
Source: Cell Calcium. Author manuscript; Available in PMC 2025 Sep 11. (PMC12424127; doi:10.1016/j.ceca.2024.102923)
Supplement: Granzotto-CellCalcium-SuppFigs [file NIHMS2102146-supplement-Granzotto-CellCalcium-SuppFigs.pdf]

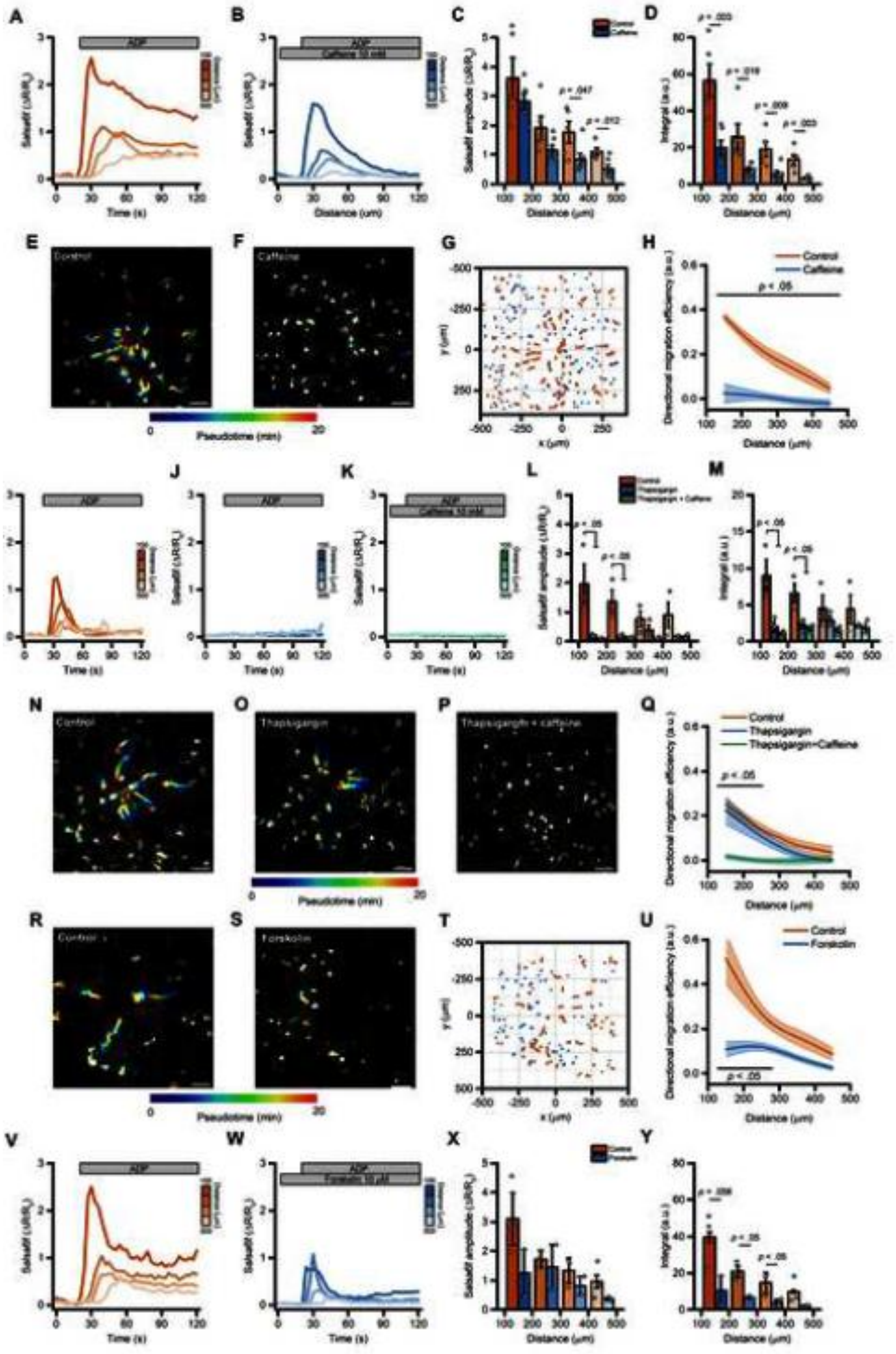

Supplementary figure 1. Directed migration of iMG cells following the generation of an ADP gradient. (A) The plot illustrates the trajectory of a representative iMG cell exposed to an ADP gradient whose maximal concentration localizes with the x = 0, y = 0 coordinates. (B) The plot depicts the line speed towards an ADP gradient of iMG cells averaged at 100 µm radial increments (n

= 4 independent experiments). (C) The plot depicts speed of ADP-treated iMG cells averaged at 100 μm radial increments (n = 4 independent experiments). Note that speed is almost constant and independent of cell distance from the gradient-generating solution. (F) The plot illustrates the trajectory of a representative iMG cell challenged as in (A) but with ADP being omitted from the gradient-generating solution. (G) The plot depicts the line speed towards the sham gradient of iMG cells averaged at 100 μm radial increments (n = 4 independent experiments). (H) The plot depicts speed of sham-treated iMG cells averaged at 100 μm radial increments (n = 4 independent experiments). (I) Pseudocolored maximum intensity projection photomicrographs of CellTracker Green CMFDA-loaded iMG differentiated from the ADRC5 iPSC line (ADRC5 iMG) and challenged with an ADP gradient. Inlet 1 shows the pseudocolored trajectory of a single ADRC5 iMG cell located in proximity to the gradient. Inlet 2 shows the pseudocolored trajectory of a single ADRC5 iMG cell located distant from the gradient. Note the almost straight pattern followed by the representative cell in Inlet 1 compared to the representative cell in Inlet 2. (J) The plot depicts directed migration efficiency towards an ADP gradient of ADRC5 iMG cells averaged at 100 μm radial increments (n = 4 independent experiments). (K) The plot depicts speed of ADP-treated ADRC5 iMG cells averaged at 100 μm radial increments (n = 4 independent experiments). (L) The plot depicts the line speed towards an ADP gradient of ADRC5 iMG cells averaged at 100 μm radial increments (n = 4 independent experiments).

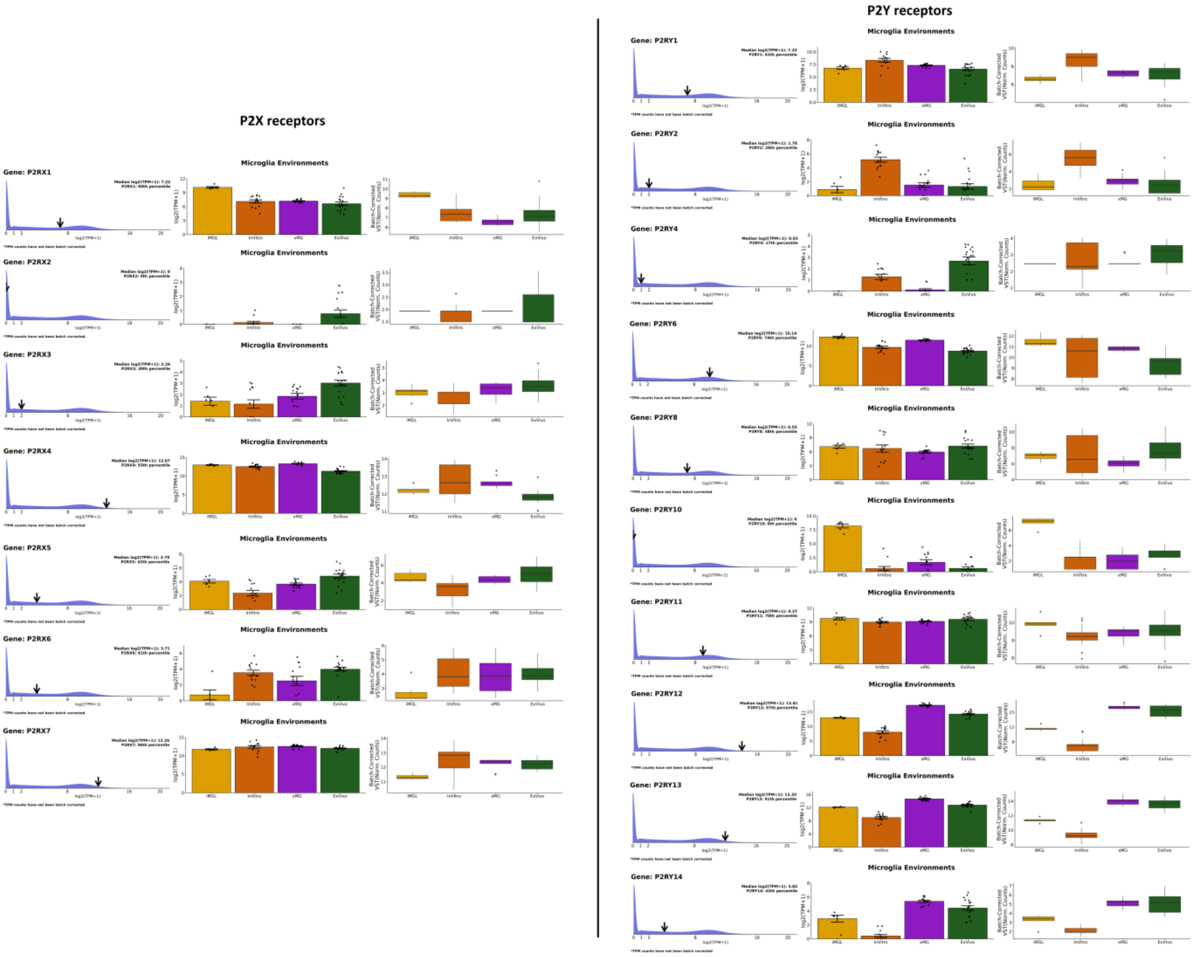

Supplementary figure 2. Purinergic receptor transcriptomic signature of iMG cells. The plots depict the expression levels of P2X and P2Y purinergic receptor families. Data were obtained from our bulk RNA-Seq database publicly available at https://rnaseq.mind.uci.edu/blurton-jones/bulkSeq/ and detailed in [39]. The left panels display density plot of gene expression across various samples; the expression level of the transcript of interest is indicated by an arrowhead. Center panels show the log2(TPM+1) expression levels of P2X and P2Y receptors in microglia form four different experimental settings: iMG cells (yellow bar), *in vitro* cultured brain-derived human microglia (InVitro; orange bar), xenotransplanted iMGs (xMG; purple bar), and *ex vivo* human microglia (ExVivo; green bar). The right most panels show box plots illustrating batch-corrected VST (variance-stabilizing transformation) normalized counts of P2X and P2Y expression in the same four microglia environments: iMGL, InVitro, xMG, and ExVivo. iMG and xMG dataset were from [39]; InVivo and ExVivo human dataset from [40]).

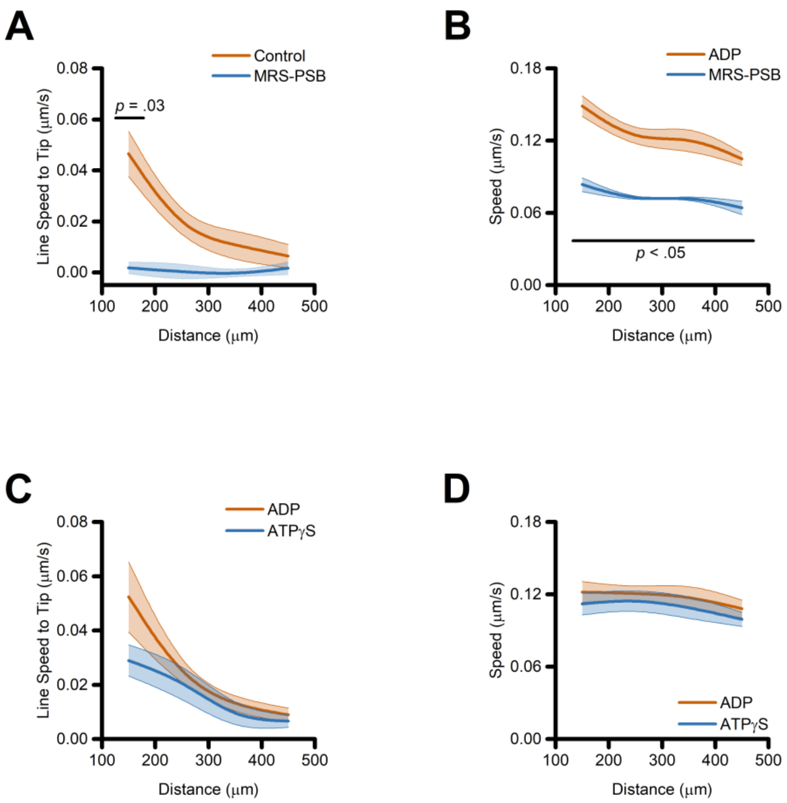

Supplementary figure 3. Directed migration of iMG cells is driven by activation of purinergic signaling. (A) The plot depicts line speed towards an ADP gradient of control and PSB 0739 + MRS 2211-treated iMG cells averaged at 100 µm radial increments (from n = 3 control and n = 2 PSB 0739 + MRS 2211 independent experiments). (B) The plot depicts speed of control and PSB 0739 + MRS 2211-treated iMG cells, challenged with ADP, and averaged at 100 µm radial increments (from n = 3 control and n = 2 PSB 0739 + MRS 2211 independent experiments). (C) The plot depicts line speed of iMG cells towards an equimolar ADP or ATPγS gradient and averaged at 100 µm radial increments (from n = 5 ADP and n = 4 ATPγS independent experiments). (D) The plot depicts speed of ADP and ATPγS -challenged iMG cells averaged at 100 µm radial increments (from n = 5 ADP and n = 4 ATPγS independent experiments). In A to D, a b-spline function was applied for curve smoothing. The comparison of mean values was assessed by a two-tailed unpaired Student's t-test.

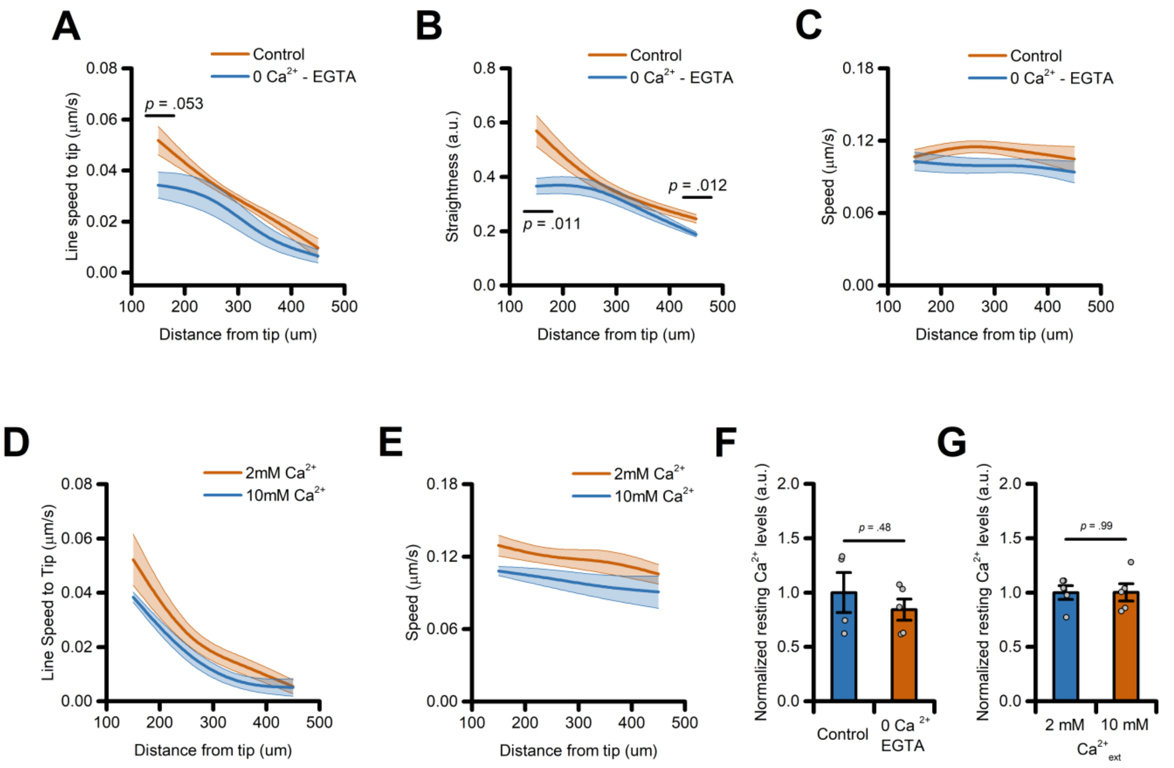

Supplementary figure 4. Directed migration of iMG cells is modestly affected by perturbation of the extracellular Ca^{2+} milieu. (A) The plot depicts directed migration efficiency towards an ADP gradient of iMG cells bathed in a control or Ca^{2+}-free medium and averaged at 100 µm radial increments (from n = 4 controls and n = 5 Ca^{2+}-free independent experiments). (B) The plot depicts straightness of iMG cells bathed in a control or Ca^{2+}-free medium, challenged with ADP, and averaged at 100 µm radial increments (from n = 4 controls and n = 5 Ca^{2+}-free independent experiments). (C) The plot depicts the speed of iMG cells bathed in a control or Ca^{2+}-free medium, challenged with ADP, and averaged at 100 µm radial increments (from n = 4 controls and n = 5 Ca^{2+}-free independent experiments). (D) The plot depicts straightness of iMG cells bathed in a control (2 mM Ca^{2+}) or 10 mM Ca^{2+}-containing medium, challenged with ADP, and averaged at 100 µm radial increments (from n = 5 controls and n = 5 10 mM Ca^{2+} independent experiments). (E) The plot depicts the speed of iMG cells bathed in a control or 10 mM Ca^{2+}-containing medium, challenged with ADP, and averaged at 100 µm radial increments (from n = 5 controls and n = 5 10 mM Ca^{2+} independent experiments). (F-G) Bar graphs depict normalized Salsa6f basal ratio values for iMG exposed to either a Ca^{2+}-free (F) or a 10mM Ca^{2+}-containing (G) extracellular medium (n = 5 independent experiment per condition). Note that the two maneuvers do not affect Ca^{2+}_i levels. In

A to E, a b-spline function was applied for curve smoothing. The comparison of mean values was assessed by a two-tailed unpaired Student's t-test.

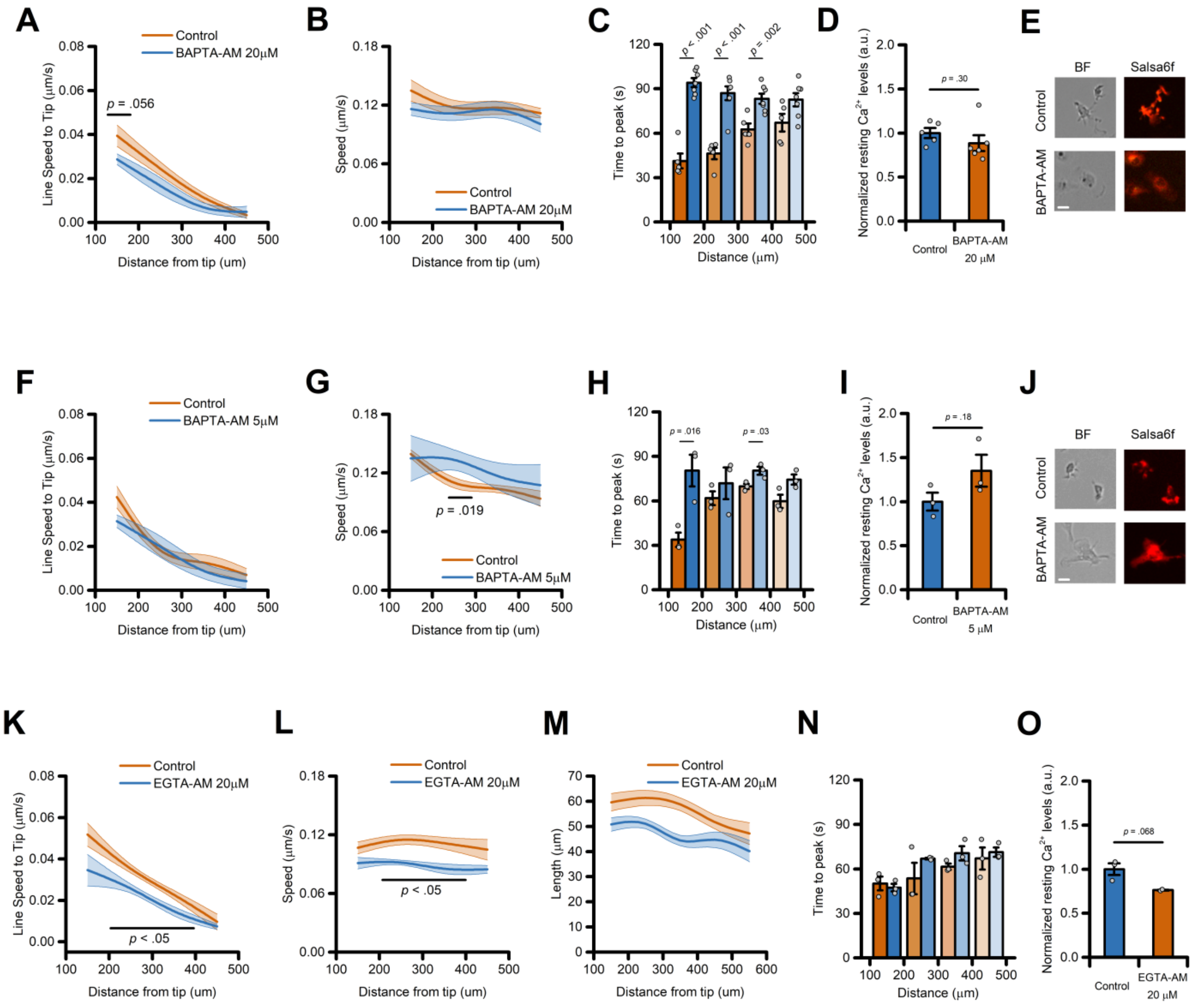

Supplementary figure 5. Directed migration of iMG cells is modestly affected by intracellular Ca^{2+} chelators. (A) The plot depicts line speed towards an ADP gradient of control and 20 µM BAPTA-loaded iMG cells averaged at 100 µm radial increments (from n = 5 control and n = 7 20 µM BAPTA independent experiments). (B) The plot depicts speed of control and 20 µM BAPTA-loaded iMG cells, challenged with ADP, and averaged at 100 µm radial increments (from n = 5 control and n = 7 20 µM BAPTA independent experiments). (C) Bar graph depicts ADP-evoked Ca^{2+}_i kinetics expressed as the time taken by a cell Ca^{2+}_i concentration to reach its maximum level (time to peak; from n = 5 control and n = 7 BAPTA 20 µM independent experiments). (D) Bar graphs depict normalized Salsa6f basal ratio values for control and 20 µM BAPTA-loaded iMG (from n = 5 control and n = 7 20 µM BAPTA independent experiments). (E) Representative brightfield (BF) and fluorescent (Salsa6f)

photomicrographs of control and 20 μM BAPTA-loaded iMG cells. Note the flattened, less branched morphology displayed by BAPTA-treated iMG cells at the end of the loading procedure. (F) The plot depicts line speed towards an ADP gradient of control and 5 μM BAPTA-loaded iMG cells averaged at 100 μm radial increments (from n = 3 control and n = 3 5 μM BAPTA independent experiments). (G) The plot depicts speed of control and 5 μM BAPTA-loaded iMG cells, challenged with ADP, and averaged at 100 μm radial increments (from n = 3 control and n = 3 5 μM BAPTA independent experiments). (H) Bar graph depicts ADP-evoked Ca^{2+}_i kinetics expressed as the time taken by a cell Ca^{2+}_i concentration to reach its maximum level (time to peak; from n = 3 control and n = 3 5 μM BAPTA independent experiments). (I) Bar graphs depict normalized Salsa6f basal ratio values for control and 5 μM BAPTA-loaded iMG (from n = 3 control and n = 3 5 μM BAPTA independent experiments). (J) Representative brightfield (BF) and fluorescent (Salsa6f) photomicrographs of control and 5 μM BAPTA-loaded iMG cells. (K) The plot depicts line speed towards an ADP gradient of control and 20 μM EGTA-loaded iMG cells averaged at 100 μm radial increments (from n = 4 control and n = 4 20 μM BAPTA independent experiments). (L) The plot depicts speed of control and 20 μM EGTA-loaded iMG cells, challenged with ADP, and averaged at 100 μm radial increments (from n = 4 control and n = 4 20 μM EGTA independent experiments). (M) The plot depicts the total length travelled by control and 20 μM EGTA-loaded iMG cells, challenged with ADP, and averaged at 100 μm radial increments (from n = 3 control and n = 3 20 μM EGTA independent experiments). (N) Bar graph depicts ADP-evoked Ca^{2+}_I kinetics expressed as the time taken by a cell Ca^{2+}_I concentration to reach its maximum level (time to peak; from n = 3 control and n = 3 20 μM EGTA independent experiments). (O) Bar graphs depict normalized Salsa6f basal ratio values for control and 20 μM BAPTA-loaded iMG (from n = 3 control and n = 3 20 μM EGTA independent experiments). In A, B, F, G, and K-M a b-spline function was applied for curve smoothing. The comparison of mean values was assessed by a two-tailed unpaired Student's t-test. Scale bars 20 μm.

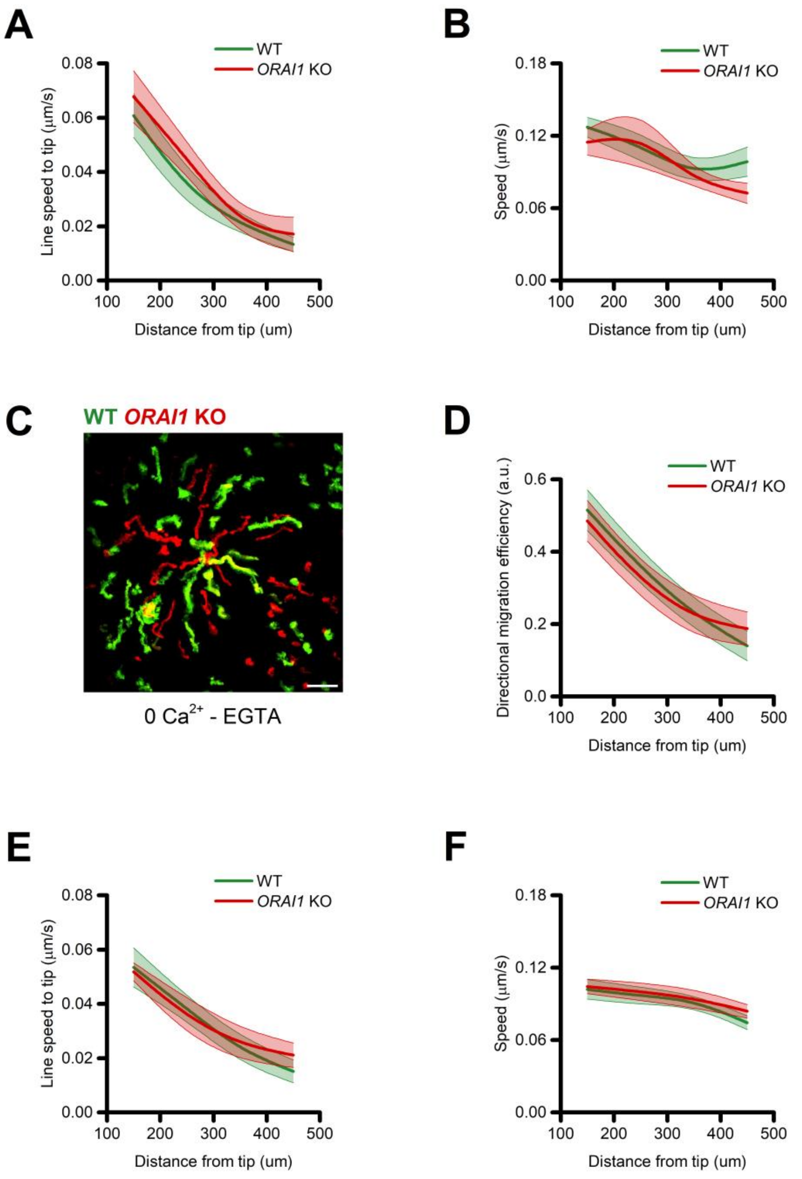

Supplementary figure 6. SOCE is not required for directed migration of iMG cells. (A) The plot depicts line speed towards an ADP gradient of WT and *ORAI1* KO iMG cells averaged at 100 µm radial increments (from n = 6 independent experiments). (B) The plot depicts speed of WT and *ORAI1* KO iMG cells, challenged with ADP, and averaged at 100 µm radial increments (from n = 6 independent experiments). (C) Maximum intensity projection photomicrographs of WT (Green) and *ORAI1* KO (Red) iMG cells challenged with an ADP gradient in Ca^{2+}-free medium. (D) The plot depicts directed migration efficiency towards an ADP gradient in Ca^{2+}-free medium of WT and *ORAI1* KO cells averaged at 100 µm radial increments (from n = 7 independent experiments). (E) The plot depicts line speed towards an ADP gradient in Ca^{2+}-free medium of WT and *ORAI1* KO cells averaged at 100

μm radial increments (from n = 7 independent experiments). (F) The plot depicts speed of WT and *ORAI1* KO iMG cells, challenged with ADP in a Ca^{2+}-free medium, and averaged at 100 μm radial increments (from n = 7 independent experiments). In A, B, and D-F a b-spline function was applied for curve smoothing. The comparison of mean values was assessed by a two-tailed unpaired Student's t-test. Scale bar 100 μm.

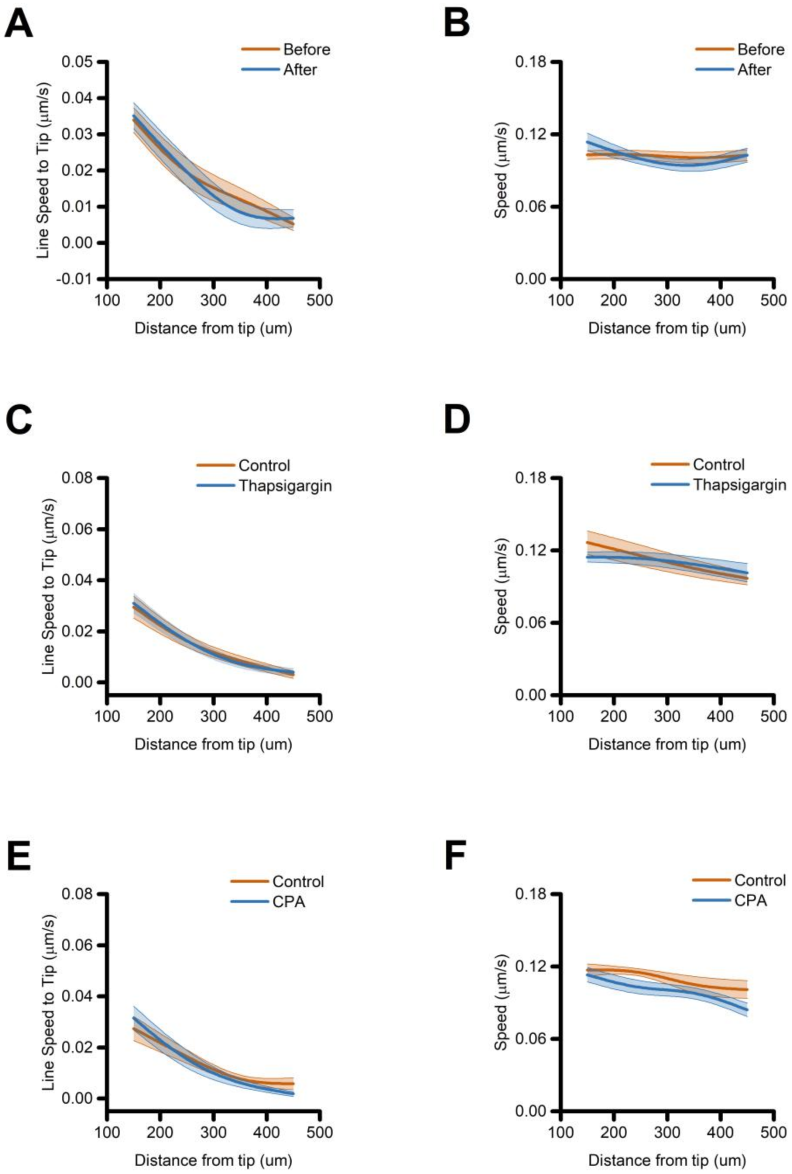

Supplementary figure 7. Ca^{2+} release from the ER is not required for directed migration in iMG.

(A) The plot depicts line speed towards an ADP gradient of iMG cells before and after ci-IP_3 uncaging

and averaged at 100 µm radial increments (from n = 7 independent experiments). (B) The plot depicts speed of iMG cells before and after ci-IP_3 uncaging, challenged with ADP, and averaged at 100 µm radial increments (from n = 7 independent experiments). (C) The plot depicts line speed towards an ADP gradient of control and 1 µM thapsigargin-treated iMG cells averaged at 100 µm radial increments (from n = 7 controls and n = 9 thapsigargin independent experiments). (D) The plot depicts speed of control and 1 µM thapsigargin-treated iMG cells, challenged with ADP, and averaged at 100 µm radial increments (from n = 7 controls and n = 9 thapsigargin independent experiments). (E) The plot depicts line speed towards an ADP gradient of control and 50 µM CPA-treated iMG cells averaged at 100 µm radial increments (from n = 6 controls and n = 7 CPA independent experiments). (F) The plot depicts speed of control and 50 µM CPA-treated iMG cells, challenged with ADP, and averaged at 100 µm radial increments (from n = 6 controls and n = 7 CPA independent experiments). A b-spline function was applied to all plots for curve smoothing. The comparison of mean values was assessed by a two-tailed unpaired Student's t-test.

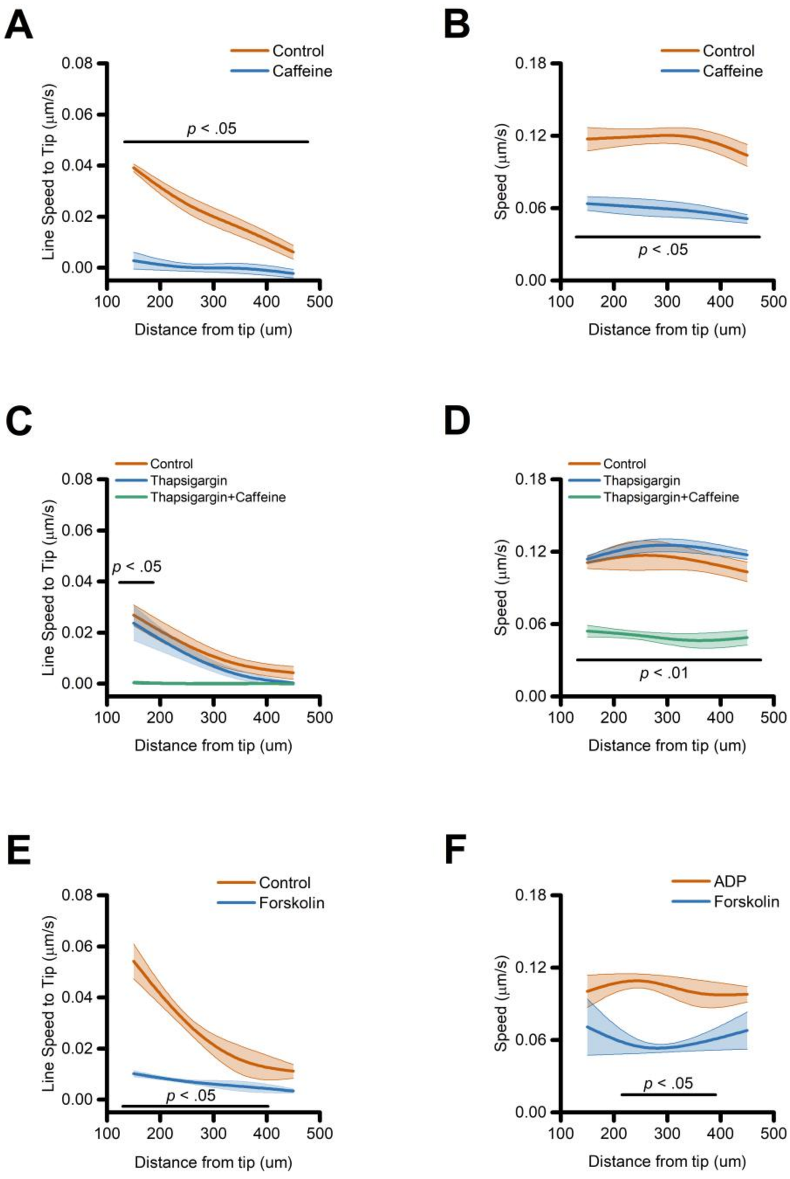

Supplementary figure 8. Directed migration of iMG cells is mediated by changes in intracellular cAMP concentrations. (A) The plot depicts line speed towards an ADP gradient of control and 10 mM caffeine-treated iMG cells averaged at 100 µm radial increments (from n = 5 controls and n = 6 caffeine independent experiments). (B) The plot depicts speed of control and 10 mM caffeine-treated iMG cells, challenged with ADP, and averaged at 100 µm radial increments (from n = 5 controls and n = 6 caffeine independent experiments). (C) The plot depicts line speed towards an ADP gradient of control, thapsigargin, and thapisgargin+caffeine-treated iMG cells averaged at 100 µm radial increments (from n = 3 controls, n = 3 thapsigargin, and n = 3 thapsigargin+caffeine independent experiments). (D) The plot depicts speed of the three populations, challenged with

ADP, and averaged at 100 µm radial increments (from n = 3 controls, n = 3 thapsigargin, and n = 3 thapsigargin+caffeine independent experiments). (E) The plot depicts line speed towards an ADP gradient of control and 10 µM forskolin-treated iMG cells averaged at 100 µm radial increments (from n = 2 controls and n = 2 forskolin independent experiments). (F) The plot depicts speed of control and 10 µM forskolin-treated iMG cells, challenged with ADP, and averaged at 100 µm radial increments (from n = 2 controls and n = 2 forskolin independent experiments). A b-spline function was applied to all plots for curve smoothing. The comparison of mean values was assessed by a two-tailed unpaired Student's t-test.

Supplementary Movie 1. The video shows a feasibility experiment demonstrating simultaneous imaging of directed migration and intracellular Ca^{2+}_i in human microglia-like cells. The video features iMG expressing the Salsa6f Ca^{2+} reporter exposed to an ADP gradient. This time series highlights the ability to monitor both Ca^{2+}_i levels and iMG dynamics over an extended period. Video acquired at 0.3 Hz. Scale bar 100 µm.

Supplementary Movie 2. The video shows a feasibility experiment for multiplex imaging of human microglia-like cell directed migration. The video exhibits two iMG populations loaded with distinct CellTracker dyes exposed to an ADP gradient. The time series illustrates the simultaneous, long-term tracking of iMG dynamics. Video acquired at 0.03 Hz. Scale bar 100 µm.
